# Full Replacement of Soybean Meal with Soybean Press Cake in Diets of Pigs Intended for Long-Cured Dry Ham Production

**DOI:** 10.3390/ani16030503

**Published:** 2026-02-05

**Authors:** Luca Sardi, Simona Belperio, Giovanna Martelli, Eleonora Nannoni

**Affiliations:** Department of Veterinary Medical Sciences, University of Bologna, 40064 Ozzano dell’Emilia, BO, Italy; luca.sardi@unibo.it (L.S.); simona.belperio2@unibo.it (S.B.); eleonora.nannoni2@unibo.it (E.N.)

**Keywords:** alternative protein source, carcass quality, pork quality, fatty acid profile, oxidative stability, PDO

## Abstract

The study investigated whether the full replacement of chemically solvent-extracted soybean meal (SM) with mechanically extracted soybean press cake (SC) could be carried out in pigs intended for dry-cured ham production. Eighty Italian heavy pigs were fattened and finished up to a body weight of 165 kg, with half being fed a commercial diet containing SM and the other half fed an isoenergetic and isonitrogenous diet in which SC fully replaced SM. Growth parameters, carcass traits, fresh meat and cured ham quality, and fatty acid composition were analyzed. Thighs were processed according to the Parma ham production rules, which require a long curing period. The only noteworthy differences observed concerned an increase in polyunsaturated fatty acid levels in the subcutaneous fat of the SC group compared with the SM group (17.26 vs. 14.58%, *p* < 0.05, in fresh thighs and 15.83 vs. 12.14%, *p* < 0.01, in cured hams), and particularly in linoleic acid (14.85 vs. 13.01%, *p* < 0.01 in fresh thighs and 13.72 vs. 10.64%, *p* < 0.01 in cured hams), which is consistent with general nutritional recommendations favoring unsaturated over saturated fatty acids. These modifications did not affect the final quality, oxidative stability, or visual appearance of the long-cured hams. In conclusion, soybean cake–based diets can be applied throughout the production cycle of heavy pigs; however, higher inclusion levels should be carefully managed, as they may affect parameters related to suitability for dry curing.

## Simple Summary

Soybean cake, produced by mechanical extraction, may represent a sustainable and novel alternative ingredient to the conventional solvent-extracted meal in pig diets. This study evaluated whether soybean press cake can fully replace soybean meal in the diets of heavy pigs used to produce traditional Italian long-cured dry hams. Eighty pigs were fed either a conventional diet or a diet in which soybean press cake completely replaced soybean meal, and their growth, carcass characteristics, and meat and ham quality were assessed. The results showed no negative effects on animal growth or on the quality of the thighs and the dry-cured hams. Pigs fed soybean press cake showed changes in fat composition, including a reduction in saturated fatty acids and an increase in polyunsaturated fatty acids, which is positive from a human nutrition standpoint. Overall, the findings indicate that soybean press cake can be used in feeding heavy pigs at amounts above the limits currently allowed for the production of traditional cured hams.

## 1. Introduction

Because of their high energy and protein content and balanced amino acid profile, soybean and its by-products have long been, and remain, the primary protein source in pig and poultry diets [1].

The most widely used by-product of soybean oil extraction (for human consumption or industrial applications) is solvent-extracted meal (commonly known simply as soybean meal, SM), which results from the industrial process of crushing soybeans and extracting the oil with the use of solvents (usually hexane) [2]. The efficiency of oil extraction is so high that, once the chemical extraction is over and the solvent is evaporated, it is possible to produce soybean meal with as little as 0.8% residual oil [3,4]. If the hulls are not added back, the defatted soybean meal contains up to 48% crude protein and no more than 1.5% oil. This resulting meal can also be referred to as a high-protein meal [5].

A much less used by-product is soybean press cake, which is obtained after oil extraction through a mechanical process (e.g., a screw press) without the addition of solvents. Due to the lower efficiency of the mechanical compared to the chemical extraction, soybean press cake (or just soybean cake, SC) is richer in oils than soybean meal, containing more than 5% residual oil [5]. Soy press cake is also a by-product of plant-based drink production, and its increased availability in recent years has led to interest in potential uses of this by-product, with the aim of avoiding the waste of useful nutrients and promoting a more circular use of this resource. Besides feedstocks, soybean press cake has also been proposed as a potential ingredient for producing meat analogs suitable for human consumption [6]. In livestock farming, its mechanical extraction process makes it an ideal by-product to be used whenever the use of feed ingredients obtained through a chemical extraction process is not allowed, such as in organic farming. The relevant legislation (Regulation EU 2018/848) in fact explicitly states that “any feed materials used or processed in organic production shall not have been processed with the aid of chemically synthesized solvents” [7], thereby making soybean press cake a potential valuable feed ingredient, especially due to the challenges in balancing the supply and requirements of essential amino acids in organic pig diets [8]. A further possible application of soybean cake in pig nutrition is in the production of typical products requiring a long dry-curing process. Parma ham, the most internationally recognized Italian Protected Designation of Origin (PDO) dry-cured ham, has recently revised its production rules to permit the inclusion of soybean press cake, which was previously not allowed, in pig diets up to 85 kg live weight, at levels of up to 10% of dietary dry matter [9]. These limitations are due to the high residual oil content of SC, which is especially rich in polyunsaturated fatty acids (PUFAs), including linoleic acid (C18:2), whose dietary intake is known to directly influence the fatty acid composition of adipose tissue and muscle in pigs [10,11]. Controlling dietary PUFA levels in the diet is therefore crucial, since, to be suitable for dry curing, the subcutaneous fat should not be excessively rich in these fatty acids. Accordingly, the production specifications set strict compositional limits for the subcutaneous fat covering the thighs: the iodine value should not be higher than 70 and/or the linoleic acid content should not exceed 15% [9]. Higher values indicate an excessive degree of unsaturation of the fat, which is associated with increased susceptibility to oxidation, negatively affecting both the processing characteristics (due to the excessive softness of the fat cover) and the sensory quality and acceptability of processed meat products (due to the development of off-flavors and a “greasy” texture) [12,13,14].

The aim of the present trial was to assess whether chemically extracted soybean meal could be completely replaced by mechanically extracted soybean cake throughout the entire growing–fattening period in heavy pigs intended for long-cured dry ham production, without impairing the technological suitability of the thighs for processing or the quality of the final product.

## 2. Materials and Methods

### 2.1. Animals, Housing, Feeding and Experimental Design

A total of 80 crossbred Duroc × (Landrace × Large White) pigs (half castrated males and half females) with an initial average body weight (BW) of approximately 50 kg were used. Pigs were raised, transported, and slaughtered in compliance with the relevant legislation [15,16,17], and they were not subjected to any invasive or painful procedures.

Animals were housed in 16 collective pens (5 pigs per pen) on fully slatted floors. Groups remained unchanged throughout the 186-day experimental period. Pigs were individually identified using ear tags and allocated homogeneously to two experimental groups (8 pens per treatment) based on their initial BW.

Each pen was equipped with a nipple drinker and a stainless-steel feeder, providing 50 cm of feeding space per pig. Artificial illumination was provided by neon tubes between 8 a.m. and 8 p.m. Environmental enrichment was provided by wood logs attached to hanging steel chains as allowed by the local regulations [18]. Pens were located in temperature-controlled rooms maintained at 22 °C and equipped with a forced-air ventilation system. Water was available ad libitum throughout the trial.

Feed was administered twice daily, at 08:30 AM and 4:30 PM. Daily feed allowances were set at 9% of the pig’s metabolic body weight (BW^0.75^), up to a maximum intake of 2.9 kg of dry matter (DM) per animal per day. The two experimental groups received different diets:

- One group was fed a traditional diet containing conventional solvent-extracted soybean meal (SM) as a protein source.
- The other group received a diet in which soybean meal was entirely replaced by mechanically extracted soybean press cake (SC).

Diets were formulated to be isoenergetic and isoproteic between the two experimental groups. As concerns protein content, the diets included soybean (meal or cake) at 15% and 12% on a dry matter basis in the growing and finishing phase, respectively. Due to the difference in crude protein (CP) content between the two protein sources (43.1% for soybean meal vs. 39.6% for soybean cake, see Table 1), additional L-lysine HCl was supplemented in the SC diet to ensure equivalent amino acid profiles between diets. Similarly, to meet the dietary energy requirements, hydrogenated palm oil was included in the first phase at different inclusion levels between the two diets. Differences in chemical and fatty acid composition among soybean meal, soybean press cake, and hydrogenated palm oil used in the study are reported in Table 1.

To meet the nutritional requirements of pigs throughout the experiment, phase-feeding was applied: one diet formulation was used during the growing phase (up to 85 kg BW) and a second one was used during the fattening–finishing phase. Diet formulation is shown in Table 2.

The acidic composition of the diets is shown in the Supplementary Materials (Table S1). The SC diets were richer in PUFAs than the SM ones. The reduced fatty acid content in the second phase is due to the lower fat content of the diets used in this phase.

### 2.2. Growth Performances

At the beginning of the study, pigs were individually weighed using a calibrated scale (DFW-03 600 kg ± 0.1, CO.BA., Bologna, Italy). Body weight (BW) was recorded on days 1, 113, and 186 of the trial to calculate the average daily gain (ADG) for each period and across the entire trial. Daily feed intake (FI) was recorded for each pen, allowing the calculation of the feed conversion ratio (FCR). Growth performance data (BW, FI, ADG, and FCR) were recorded until day 186, when at least 50% of the pigs had achieved the intended BW (approximately 165 kg). Animals were slaughtered in two batches, separated by a 15-day interval.

### 2.3. Carcass Traits and Fresh Meat Quality

Following a 12 h feed withdrawal, animals were slaughtered under commercial conditions. Carcasses were weighed, and carcass composition was estimated with a Fat-o-Meater (SFK, Copenhagen, Denmark). Muscle pH was measured at 45 min post-mortem in both the Longissimus dorsi (in the region between the 4th and the 6th rib) and the Semimembranosus muscles using a portable pH meter (model 250A; Orion Research, Boston, MA, USA). A second pH measurement (pH 24 h) was performed in the Semimembranosus muscle at 24 h post-mortem. After carcass dissection, the main commercial cuts (loin and thigh) were weighed. Instrumental color (L*, a*, and b*) was measured with a Konica Minolta CR-400 colorimeter (Minolta Camera Company, Osaka, Japan) using D65 illuminant, 2° observer angle, and an 8 mm aperture on the Gluteus medius of the ham.

Immediately after carcass dissection, two 20 mm thick samples (150 g) were cut from the posterior end of the Longissimus dorsi muscle from each half carcass. The samples were individually packaged and transported under refrigerated conditions (4 °C) to the laboratory. On the first sample, drip loss and cooking loss were determined following the standard procedure described by Honikel [19] and Sardi et al. [20]. On the second sample, chemical (moisture, crude protein, intramuscular fat, and ash) and fatty acid composition were performed according to standard AOAC Official Methods [21].

The day after slaughter, the refrigerated thighs were trimmed at the slaughterhouse to remove excess fat, muscle, and skin. On that occasion, a meat sample was collected from the Semimembranosus muscle, and a subcutaneous fat sample was collected from the area overlying the Biceps femoris. Both samples were transferred to the lab for the subsequent determination of the chemical composition (of the meat sample, according to the methods recalled above) and of the fatty acid composition (of both the lean and the fat samples).

To assess the fatty acid composition, total lipids from Longissimus dorsi and Semimembranosus were extracted using the Folch method [22], and fatty acid content was evaluated using gas chromatography (HRGC8560 Series Mega 2gas chromatograph; Fisions Instruments, Milan, Italy). Fatty acids were esterified with 5% methanolic hydrogen chloride. The fatty acid methyl esters were separated by gas chromatography using a Supelco SP-2330 capillary column (length: 30 m; internal diameter: 0.25 mm; film thickness: 0.2 μm; Supelco, Bellefonte, PA, USA). The iodine value, used as an indicator of lipid unsaturation, was determined according to the AOAC Official Method [21].

### 2.4. Quality and Appearance Assessment of Hams

The dry-curing process was conducted in accordance with the Parma Ham specifications [9], which provide a standardized technological framework for high-quality, dry-cured ham production. According to these rules, thighs are first salted for approximately 25 days and subsequently rested for a maximum of 90 days in refrigerated, humidity-controlled rooms. Hams are then hung in well-ventilated rooms for three months; after this period, the exposed muscular surface is greased, and hams are transferred to the cellars (cooler darker and less ventilated rooms) and hung on racks until the dry curing is completed (minimum 14 months from salting). In the present study, 32 right thighs per group were monitored throughout a 24-month dry-curing process. Weight was recorded at each major stage to evaluate weight loss over time: before and after trimming, after salting, and at the end of the dry-curing period. At the end of the dry-curing process, when hams were deboned, 32 hams (16 per group) were sampled. To this aim, a slice (including the Biceps femoris and Semimembranosus muscles) was taken transversally, from the caudal portion of the ham to the middle of the femoral bone impression. A panel of 4 trained experts visually evaluated each sample according to Sardi et al. [23], expressing a score on a scale from 1 to 10 (where 1 = absence of the trait; 10 = maximum presence) for parameters of the lean (texture, color inhomogeneity, and marbling) and of the fatty (texture and thickness) portions. The overall sensory score of the ham was rated on a scale ranging from 1 to 10 (where 1 = very bad quality; 10 = optimal characteristics) as reported by Nannoni et al. [24].

Samples of the Biceps femoris muscle from cured hams were collected for chemical analyses, including moisture content and crude protein according to AOAC methods [21], sodium chloride (NaCl) concentration, and proteolysis index according to previously published methods [25,26]. In addition, subcutaneous fat samples (outer and inner layers) from the overhanging region of the Biceps femoris muscle were taken to determine fatty acid composition following the method described above, as well as peroxide value and specific UV extinction coefficients K232 (K_1_) and K268 (K_2_) according to AOAC methods [21], and thiobarbituric acid reactive substances (TBARS) [27], as indicators of lipid oxidation. All analyses were carried out one week after the sampling.

### 2.5. Statistical Analyses

Sample size was calculated using G*Power software (version 3.1, University of Dusseldorf, Düsseldorf, Germany) [28]. For sample size estimation, fat quality parameters were prioritized. Specifically, a potential variation of 1 percentage point in linoleic acid content (C18:2) and a variation of 4 points in iodine value were considered. Assuming a statistical power (1−β) of 80% and a significance level of α = 0.05, the resulting sample size was 40 samples in total (20 per group).

Statistical analyses were performed using Statistica software (version 10.0; StatSoft Inc., Tulsa, OK, USA). All parametric data were analyzed using the General Linear Model (GLM) procedure, with the experimental group (i.e., diet) and sex as the main fixed effects. Initially, the slaughtering batch was also considered in the model; however, it was later removed due to the absence of statistically significant effects. The experimental unit was the pen for growth and carcass traits, and the individual for meat and ham quality parameters. Sensory data from the panel test were analyzed by the non-parametric Mann–Whitney U test. Results are presented as group mean and Error Mean Square (EMS) for all parametric analyses. For the sensory evaluation, results are reported as mean values accompanied by the standard error (SE). As the sex did not result in any statistically significant effects, data are reported by experimental group only. Differences in mean values were considered statistically significant at *p* ≤ 0.05.

## 3. Results

### 3.1. Animal Health and Growth Performances

Throughout the trial, pigs were checked daily for their health status. No health issues were observed, no veterinary treatments or clinical interventions were necessary, and all animals completed the trial as planned.

No statistically significant differences were observed between the SM and SC groups for BW at any time point, ADG, or FCR throughout the study (see Table S2 in Supplementary Materials).

### 3.2. Carcass and Meat Quality

As shown in Table 3 and Table 4, no statistically significant differences were observed between pigs fed SM and SC diets in terms of carcass characteristics or meat quality parameters (including pH, instrumental color, and water holding capacity).

Table 5 shows the proximate composition of the Longissimus dorsi and Biceps femoris muscles in the two experimental groups. No significant differences were detected for the parameters analyzed at the Longissimus dorsi level, whereas in the Biceps femoris, a higher moisture content was observed in the SC group (*p* = 0.02), resulting in a significant reduction in protein content (*p* = 0.05).

### 3.3. Fatty Acid Composition of Intramuscular and Subcutaneous Fat of the Raw Meat

The fatty acid composition of the intramuscular fat from the loins was very similar between the groups (Table 6). The most common saturated fatty acids (SFAs) were C16:0 and C18:0, and the overall SFA content was similar between groups. Among monounsaturated fatty acids (MUFAs), C18:1 was the most prevalent, followed by C16:1. Polyunsaturated fatty acids (PUFA) were mainly represented by C18:2, followed by C20:4. The latter, arachidonic acid, showed higher values in the SC group (*p* = 0.05). However, the overall PUFA, MUFA, and SFA contents were not different between treatments.

The effects of the diet on the fatty acid composition of the subcutaneous fat covering the thighs were more pronounced (Table 7). The SM group had greater proportions of C14:0, C16:0, and C18:0 than SC (*p* < 0.05 for all variables), resulting in a significantly higher overall SFA content (*p* = 0.01). Among MUFAs, no differences were observed in the single fatty acids or in overall MUFA content. Differences in PUFAs were more pronounced, with SC having higher C18:2, C18:3, and C20:4 levels than SM, resulting in a significantly higher overall PUFA content (*p* < 0.05 for all differences). As a consequence of the increased unsaturation degree, iodine number was also significantly higher in SC than in SM (*p* = 0.01).

### 3.4. Weight Losses and Quality of Dry-Cured Hams

No significant differences were observed between groups as concerns ham weight losses during the dry-curing stages (Table 8). In addition, the subcutaneous fat thickness and the overall weight loss during processing were unaffected by diet.

The ham quality parameters revealed no significant differences between groups in proximate composition, salt content, or color of the lean and fat portions (Table 9). Only the proteolysis index was affected by the dietary treatment (*p* = 0.03), with hams from SC having a lower proteolysis index than those from SM.

### 3.5. Fatty Acid Composition, Oxidative Stability, and Appearance of Cured Hams

Also, the overall fatty acid composition of the intramuscular fat in the Biceps femoris from the cured thighs was unaffected by the dietary treatment (Table 10). Only tendential differences (*p* < 0.1) were observed, with palmitoleic acid (C16:1) being higher in SM and linoleic acid (C18:2) being higher in SC.

Table 11 shows the fatty acid composition of the subcutaneous fat of the thigh after curing. Cured hams from the SM diet had significantly higher proportions of most SFAs and MUFAs than SC, including myristic (C14:0), palmitic (C16:0), and palmitoleic (C16:1) acid (*p* < 0.01 for all differences). C18:1 (oleic acid) was also tendentially higher in SM than in SC. Conversely, the SC diet resulted in increased amounts of PUFAs, particularly linoleic (C18:2), linolenic (C18:3), and arachidonic (C20:4) acids, leading to a greater total PUFA content (*p* ≤ 0.01 for all differences). Ham lipid oxidation indicators, such as the peroxide value and specific extinction coefficient, measured at 232 and 268 nm, and TBARS showed no significant differences between the two dietary regimens.

Lastly, no visually discernible differences were observed between the two groups in the cured hams (Table 12), either for individual traits or for the overall score.

## 4. Discussion

Overall, our results are consistent with other studies on Italian heavy pigs slaughtered at similar weights, both in terms of production parameters and ham quality [23,24,29,30,31] and supporting the view that solvent-extracted soybean meal can be successfully replaced by alternative protein sources when pig diets are properly balanced for amino acid profile and energy supply [32]. In addition, the technological properties of fresh meat remained unaffected by the dietary treatment, with all values falling within the expected range for thighs intended for long dry curing [23,33].

With respect to fresh meat, the only significant variation observed was a slightly higher, although significant, moisture content in the thigh muscle of the animals fed soybean cake. However, as this increase was not associated with changes in other technological or qualitative parameters of either fresh or dry-cured thighs, it does not appear to be of practical relevance. Concerning the fatty acid composition of lean and subcutaneous fat, results from the present study are in line with those observed in younger pigs by Świątkiewicz et al. [34], who examined the complete replacement of solvent-extracted soybean meal with extruded soybean press cake in weaned piglets (initial body weight of 9 kg) fed isoenergetic and isonitrogenous diets. At slaughter (carried out at 35 days of age and 34 kg BW), samples of Longissimus dorsi and subcutaneous backfat were collected for fatty acid analysis. The authors reported that the substitution had no significant effect on growth performance or feed consumption, and no adverse consequences were observed in intestinal morphology or metabolic health indicators. Similarly to the present results obtained in heavy pigs, an increase in the proportion of polyunsaturated fatty acids was observed in both muscle and adipose tissue; however, lipid oxidative stability, evaluated using the thiobarbituric acid reactive substances method, was not affected.

In our study, the main effect of the soybean cake diet was a shift in the fatty acid profile of the pig tissues. Specifically, in the intramuscular fat extracted from the loin, arachidonic acid (C20:4) was significantly increased compared to the soybean meal diet. In the subcutaneous fat covering the fresh thighs, the overall SFA content was significantly reduced, whereas PUFAs were significantly increased. This variation in the fatty acid composition was expected and is consistent with the fatty acid profile of the experimental diets. It is well established that the fatty acid composition of animal adipose tissue closely reflects dietary lipid intake, with diets rich in polyunsaturated fatty acids leading to a less saturated fat profile, and vice versa, whereas intramuscular fat is comparatively less responsive to dietary manipulation [35,36]. For this reason, limits for specific fatty acid contents (in particular for linoleic acid, C18:2) are established in diets for heavy pigs intended for dry-cured ham production (e.g., [9]) in order to preserve the technological properties of the raw thighs and avoid risks of reduced fat texture and excessive lipid oxidation during the long dry-curing process.

The relationship between dietary lipid composition and tissue fatty acid profile can also be deliberately exploited to modulate the fatty acid composition of pork products with the aim of improving their nutritional quality and the resulting ω-6/ω-3 ratio [37,38]. In the present study, however, the ω-6/ω-3 ratio varied depending on the tissue considered (intramuscular fat and subcutaneous fat). Nevertheless, when considered overall, the changes observed in the subcutaneous fat fatty acid profile of pigs fed the soybean cake diet, and, in particular, the increase in linoleic acid (C18:2 ω-6), linolenic acid (C18:3 ω-3) and arachidonic acid (C20:4 ω-6), were accompanied by a consistent reduction in SFAs, resulting in a generally favorable balance from a human nutritional perspective [39]. It is worth noting that the suitability of the fat for dry curing was assessed in a random sample of hams from different batches at the abattoir. Batch compliance was established according to a predefined acceptance threshold for non-compliant results (e.g., 25%). In our study, the risk of having samples above the threshold values (C18:2 > 15% and iodine value > 70) slightly increased in the SC group (from 4 to 12% for linoleic acid and from 0 to 5% for the iodine value); therefore, the SC batch was compliant with the requirements. It is also worth noting that the overall variation in lipid composition observed (namely the reduction in SFAs and the increase in PUFAs), while resulting in a significantly higher iodine value, did not impact the suitability of the thighs for the curing process, as both linoleic acid and the iodine value remained below the thresholds defined by the Parma Ham production rules (≤15% C18:2; iodine value ≤ 70). However, since the linoleic acid values in the SC group approached the upper limit, there might be a need for careful monitoring of total linoleic acid dietary content when soybean cake is used at high inclusion levels. Weight loss during curing and the major macroscopic characteristics of hams were not affected by the dietary treatment. Overall values aligned with previous studies on the quality of Italian cured ham [23,24]. The only significant difference between the experimental groups was a slightly lower proteolysis index in group SC, which approached the minimum limit of acceptability set by the production rules. Nevertheless, cured hams met the quality requirements in terms of weight (11.8–18.0 kg), moisture (58–63%), and proteolysis (25–32%) [9]. In both experimental groups, the salt content measured at the end of the dry-curing period exceeded the range established by the production rules (4.2–6.0%) [9]. However, this threshold refers to hams aged for 14 months, whereas in the present study, analyses were performed after 24 months of ripening. The extended duration of the curing process may therefore be responsible for the higher sodium chloride concentration observed. In addition, it has been reported that weight losses and salt penetration are mainly determined by intrinsic muscle characteristics and curing technology rather than by dietary changes [35], further supporting the lack of a diet-related effect on salt content in the present study. In cured hams, the fatty acid profile of the intramuscular fat did not appear to be influenced by the different protein sources, while the subcutaneous fat mirrored the differences already observed in the raw thighs, with lower proportions of saturated and monounsaturated fatty acids and higher levels of polyunsaturated fatty acids in pigs fed the soybean cake diet. Notwithstanding the higher degree of unsaturation, none of the lipid oxidation parameters differed significantly between treatments, suggesting that oxidative stability was not adversely affected.

The absence of statistically significant differences related to sex in fatty acid composition agrees with previous findings [40]. On the contrary, Lo Fiego et al. [41] found some sex-related differences, although they highlight that other factors, such as body weight at slaughter, are much more impactful. As concerns fat from cured hams, Toscano et al. [42] found only minimal sex-related variations in fatty acid composition.

Lastly, the absence of differences in the visual appearance of the cured hams suggests that the complete replacement of soybean meal with soybean cake was not associated with measurable changes in consumer-perceived quality.

## 5. Conclusions

The complete replacement of soybean meal with soybean cake in the diet of heavy pigs intended for dry-cured ham production did not appear to affect growth performance, fresh meat quality (including the suitability of the thighs for the dry-curing process), or the quality of cured hams. The fatty acid composition of the subcutaneous fat, both in raw and cured thighs, showed an increase in polyunsaturated fatty acids, particularly linoleic acid, together with a reduction in saturated fatty acids, a modification that is generally considered consistent with current nutritional recommendations.

Based on these findings and under the experimental conditions of the present study, soybean press cake may be considered suitable for use in diets for heavy pigs throughout the entire production cycle, without evidence of detrimental effects on fresh or processed meat.

Nevertheless, should the use of soybean press cake be extended to the entire production cycle of pigs intended for PDO ham production, careful monitoring of dietary PUFA levels would be advisable, particularly when inclusion rates of soybean press cake during the finishing phase approach 12%, in order to ensure full compliance with ham quality standards.

## Figures and Tables

**Table 1:** Chemical and fatty acid composition of soybean meal, soybean cake, and hydrogenated palm oil used in the diet formulation.

	Soybean Meal	Soybean Cake	Hydrogenated Palm Oil
Chemical composition (% as is)			
Dry matter	88.41	93.82	98.2
Crude protein	43.12	39.56	-
Ether extract	1.60	6.52	96.5
Crude fiber	8.37	5.62	-
Ash	6.45	5.98	
Fatty acid composition (g/kg)			
C 14:0	0.2	0.2	19.2
C 16:0	1.3	8.0	441.8
C 18:0	0.5	2.7	297.3
C18:1	2.8	16.5	234.7
C 18:2	6.8	38.7	-
C 18:3	0.9	5.2	-
SFAs	2.0	10.9	758.3
MUFAs	2.8	16.5	234.7
PUFAs	7.7	43.9	-

SFAs = saturated fatty acids; MUFAs = monounsaturated fatty acids; PUFAs = polyunsaturated fatty acids.

**Table 2:** Composition and chemical analysis of experimental diets in the growing and finishing phases.

	Growing Phase <85 kg BW		Finishing Phase >85 kg BW—End	
Groups	SM	SC	SM	SC
Ingredients (%)				
Maize meal	45.0	45.0	49.4	49.4
Barley meal	16.55	16.6	20.0	20.0
Soybean meal, S.E.	15.0	-	12.0	-
Soybean cake	-	15.0	-	12.0
Soft wheat bran	13.5	13.68	10.47	10.45
Faba bean meal	5.0	5.0	5.0	5.0
Hydrogenated palm oil	1.6	1.35	-	-
Mineral–vitamin premix	3.25	3.25	3.05	3.05
Lysine	0.1	0.12	0.08	0.1
Chemical composition (on a DM basis)				
Analyzed values				
Crude protein (%)	16.55	16.29	15.32	15.10
Ether extract (%)	4.73	5.24	3.03	3.65
Crude fiber (%)	5.26	5.16	5.00	4.90
Ash (%)	6.30	6.28	5.89	5.87
Linoleic acid (%)	1.42	1.96	1.46	1.89
Calculated values				
Digestible energy (kcal/kg)	3669	3681	3621	3639
Net energy (kcal/kg)	2653	2698	2620	2670
Lysine (%)	0.91	0.90	0.81	0.80
Calcium (%)	0.97	0.96	0.89	0.89
Phosphorus (%)	0.67	0.67	0.61	0.61

DM = dry matter; SM = soybean meal; SC = soybean cake.

**Table 3:** Carcass traits.

Groups		SM	SC	EMS	*p*-Value
Pens	n.	8	8	-	
Carcass weight (CW)	kg	140.7	141.9	47.2	0.73
Carcass yield	%	81.9	81.8	0.7	0.76
F-o-M lean meat	%	49.7	50.1	2.2	0.62
Loin thickness	mm	61.5	61.9	13.6	0.83
Fat thickness	mm	25.3	24.5	7.4	0.60
Loin	% of CW	10.8	10.7	0.28	0.68
Thigh	% of CW	25.6	25.5	0.21	0.56
ph45’ loin		6.32	6.26	0.03	0.44
pH45’ thigh		6.19	6.11	0.007	0.10
pH24 h thigh		5.61	5.64	0.001	0.24

SM = soybean meal; SC = soybean cake; EMS = Error Mean Square.

**Table 4:** Meat qualitative traits (color of Semimembranonsus muscle and water holding capacity of Longissimus dorsi muscle).

Groups	SM	SC	EMS	*p*-Value
Samples (n.)	24	24	-	
L*	50.5	50.8	6.43	0.72
Hue	0.78	0.77	0.01	0.71
Chroma	9.2	9.0	2.41	0.63
Drip loss (%)	3.31	3.42	0.57	0.82
Cooking loss (%)	20.21	20.46	9.97	0.68

SM = soybean meal; SC = soybean cake; EMS = Error Mean Square.

**Table 5:** Chemical compositions of loin (Longissimus dorsi) and thigh lean tissue (Biceps femoris).

Groups		SM	SC	EMS	*p*-Value
Longissimus dorsi					
Samples (n.)		24	24	-	
Humidity (%)	%	73.7	73.8	1.1	0.50
Crude protein (%)	“	20.8	20.8	0.67	0.68
Ether extract (%)	“	4.4	4.3	2.51	0.53
Ash (%)	“	1.1	1.1	0.004	0.46
Biceps femoris					
Samples (n)		24	24	-	
Humidity (%)	%	72.4	73.0	1.0	0.02
Crude protein (%)	“	23.7	23.3	0.40	0.05
Ether extract (%)	“	2.8	2.5	1.1	0.33
Ash (%)	“	1.2	1.2	0.004	0.89

SM = soybean meal; SC = soybean cake; EMS = Error Mean Square.

**Table 6:** Fatty acid composition of the intramuscular fat from the loin (data are expressed as percentages of each fatty acid).

Groups	SM	SC	EMS	*p*-Value
Samples (n.)	24	24		
C 14:0	1.03	0.99	0.07	0.93
C 16:0	21.13	20.61	1.22	0.82
C 16:1	3.33	3.26	0.43	0.22
C 18:0	9.86	9.79	1.12	0.96
C 18:1	45.75	46.01	9.01	0.46
C 18:2	10.80	11.41	4.07	0.38
C 18:3	0.17	0.14	0.04	0.55
C 20:4	1.74	1.94	0.24	0.05
SFAs	32.37	31.75	3.54	0.95
MUFAs	54.80	54.62	9.13	0.31
PUFAs	12.83	13.63	5.18	0.20

SM = soybean meal; SC = soybean cake; EMS = Error Mean Square; SFAs = saturated fatty acids; MUFAs = monounsaturated fatty acids; PUFAs = polyunsaturated fatty acids.

**Table 7:** Fatty acid composition and iodine number of the subcutaneous fat of the thigh (data are expressed as percentages of each fatty acid).

Groups	SM	SC	EMS	*p*-Value
Samples (n.)	24	24		
C 14:0	1.75	1.49	0.04	0.01
C 16:0	23.31	22.05	1.07	0.01
C 16:1	2.13	1.81	0.10	0.06
C 18:0	12.29	11.74	0.92	0.02
C 18:1	43.25	42.75	3.83	0.70
C 18:2	13.01	14.85	3.32	0.01
C 18:3	0.64	0.96	0.03	0.01
C 20:4	0.65	0.77	0.02	0.02
SFAs	38.22	36.23	2.29	0.01
MUFAs	47.20	46.52	7.26	0.64
PUFAs	14.58	17.26	9.64	0.02
Iodine number	62.46	66.7	13.3	0.01

SM = soybean meal; SC = soybean cake; EMS = Error Mean Square; SFAs = saturated fatty acids; MUFAs = monounsaturated fatty acids; PUFAs = polyunsaturated fatty acids.

**Table 8:** Weight losses of hams during the dry-curing process.

Groups		SM	SC	EMS	*p*-Value
Hams	n.	32	32		
Weight of the hot thigh	kg	17.25	17.75	3.13	0.32
Weight of the cold thigh	kg	16.98	17.42	2.94	0.36
Weight loss after chilling	%	1.53	1.84	1.30	0.33
Subcutaneous fat thickness	cm	2.77	2.87	0.15	0.58
Weight of the trimmed thigh	kg	13.83	14.14	1.52	0.38
Trimming weight loss	%	18.38	18.70	7.24	0.57
Weight after salting	kg	13.51	13.81	1.46	0.40
Salting weight loss	%	2.32	2.36	1.15	0.88
Cured weight (24 months)	kg	9.69	10.01	1.15	0.24
Total weight loss	%	30.12	29.27	5.67	0.10

SM = soybean meal; SC = soybean cake; EMS = Error Mean Square.

**Table 9:** Quality of dry-cured hams.

Groups		SM	SC	EMS	*p*-Value
Samples	n.	16	16	-	
Moisture	%	60.82	60.91	2.8	0.88
Crude protein	%	27.49	27.22	0.56	0.32
Proteolysis index	%	26.68	25.10	3.73	0.03
Salt (NaCl)	%	6.61	6.69	0.25	0.63
Color (Biceps femoris muscle)					
L*		34.83	35.74	4.54	0.24
Hue		0.34	0.38	0.01	0.21
Chroma		8.58	8.62	3.02	0.95
Color (subcutaneous fat)					
L*		70.83	70.39	3.30	0.50
Hue		−1.35	−1.34	0.01	0.79
Chroma		6.81	6.90	0.33	0.64

SM = soybean meal; SC = soybean cake; EMS = Error Mean Square.

**Table 10:** Fatty acid composition of the intramuscular fat of the lean tissue (Biceps femoris) of dry-cured hams (data are expressed as percentages of each fatty acid).

Groups	SM	SC	EMS	*p*-Value
Samples (n.)	16	16	-	
C 14:0	0.98	0.97	0.06	0.86
C 16:0	20.35	20.04	1.20	0.43
C 16:1	3.40	2.97	0.37	0.06
C 18:0	9.54	9.47	0.97	0.85
C 18:1	44.29	44.48	8.42	0.85
C 18:2	10.15	11.34	3.54	0.09
C 18:3	0.16	0.14	0.04	0.72
C 20:4	1.86	1.70	0.25	0.38
SFAs	32.13	31.80	3.60	0.63
MUFAs	54.81	54.18	9.09	0.56
PUFAs	13.06	14.02	5.24	0.25

SM = soybean meal; SC = soybean cake; EMS = Error Mean Square; SFAs = saturated fatty acids; MUFAs = monounsaturated fatty acids; PUFAs = polyunsaturated fatty acids.

**Table 11:** Fatty acid composition and oxidative stability of the subcutaneous fat of dry-cured hams (data are expressed as percentages).

Groups	SM	SC	EMS	*p*-Value
Samples (n.)	16	16		
C 14:0	1.51	1.39	0.01	0.01
C 16:0	21.97	20.75	0.52	0.01
C 16:1	2.43	1.99	0.14	0.01
C 18:0	10.33	10.08	0.59	0.37
C 18:1	45.58	44.01	4.85	0.06
C 18:2	10.64	13.72	4.39	0.001
C 18:3	0.63	0.95	0.02	0.001
C 20:4	0.60	0.76	0.01	0.001
SFAs	34.56	33.09	0.96	0.001
MUFAs	53.30	51.08	4.38	0.01
PUFAs	12.14	15.83	5.26	0.001
Peroxides (meq O_2_/kg)	9.24	10.67	10.31	0.22
K1 (232 nm)	2.51	2.46	0.20	0.78
K2 (268 nm)	0.11	0.10	0.01	0.31
TBARS (MDA mg/kg)	1.36	1.33	0.28	0.88

SM = soybean meal; SC = soybean cake; EMS = Error Mean Square; SFAs = saturated fatty acids; MUFAs = monounsaturated fatty acids; PUFAs = polyunsaturated fatty acids; K1 and K2 = specific extinction coefficient measured at 232 and 268 nm, respectively; TBARS = thiobarbituric acid reactive substances; MDA = Malondialdehyde.

**Table 12:** Appearance assessment of dry-cured hams (Data are expressed on a 1 to 10 scale: 1 = absence of the trait; 10 = maximum presence. For the overall score: 1 = very bad quality; 10 = optimal characteristics).

Groups	SM	SC	SE	*p*-Value
Samples (n.)	16	16		
Lean portion				
Wet Surface	1.4	1.9	0.19	0.17
Texture	5.3	5.4	0.17	0.64
Color inhomogeneity	2.6	2.5	0.23	0.90
Marbling	0.3	0.1	0.07	0.56
Fat portion				
Texture	4.4	4.8	0.17	0.34
Thickness	3.4	3.9	0.29	0.64
Oily surface	2.2	2.2	0.13	1.0
Overall score	6.4	6.4	0.33	0.96

SM = soybean meal; SC = soybean cake; SE = standard error.

## Data Availability

The original data presented in the study are openly available in https://amsacta.unibo.it/id/eprint/8771 (accessed on 26 January 2026).

## Supplementary Materials

The following supporting information can be downloaded at https://www.mdpi.com/article/10.3390/ani16030503/s1. Table S1. Acidic composition of the experimental diets used in the growing and finishing phases, expressed as g/kg of dry matter. The growing phase included pigs with body weight < 85 kg, whereas the finishing phase included pigs with body weight > 85 kg until slaughter. Diets were based on soybean meal (SM) or soybean cake (SC). SM = Soybean Meal; SC = Soybean Cake; BW = Body Weight; SFA = Saturated Fatty Acids; MUFA = Monounsaturated Fatty Acids; PUFA = Polyunsaturated Fatty Acids. Table S2. Growth performance of growing–finishing pigs fed diets based on soybean meal (SM) or soybean cake (SC). Data are reported as group means; pens represent the experimental units. SM = Soybean Meal; SC = Soybean Cake; BW = Body Weight; ADG = Average Daily Gain; FCR = Feed Conversion Ratio; EMS = Error Mean Square.

## Abbreviations

The following abbreviations are used in this manuscript:

SM	Soybean Meal (solvent-extracted)
SC	Soybean Cake (mechanically extracted)
DM	Dry Matter
BW	Body Weight
ADG	Average Daily Weight Gain
FCR	Feed Conversion Rate
SFAs	Saturated Fatty Acids
MUFAs	Monounsaturated Fatty Acids
PUFAs	Polyunsaturated Fatty Acids
TBARS	Thiobarbituric Acid Reactive Substances
MDA	Malondialdehyde
EMS	Error Mean Square
SE	Standard Error
PDO	Protected Designation of Origin
