# Supplementary material for: Full Replacement of Soybean Meal with Soybean Press Cake in Diets of Pigs Intended for Long-Cured Dry Ham Production
Source: Animals (Basel). 2026 Feb 5;16(3):503. doi: 10.3390/ani16030503 (PMC12896938; doi:10.3390/ani16030503)
Supplement: Supplementary file 1 [file animals-16-00503-s001.zip › animals-4105036-supplementary.pdf]

Table S1: Acidic composition of the experimental diets used in the growing and finishing phases. expressed as g/kg on the dry matter

	**Growing phase <85kg BW**		**Finishing phase >85 kg BW – end**	
**Groups**	**SM**	**SC**	**SM**	**SC**
C14:0	0.38	0.36	0.04	0.07
C16:0	11.39	11.28	3.47	4.37
C16:1	0.07	0.07	0.07	0.07
C18:0	5.84	5.38	0.48	0.79
C18:1	10.12	11.78	6.10	7.96
C18:2	14.20	19.61	14.62	18.91
C18:3	0.72	1.45	0.67	1.26
C20:4	0.06	0.06	0.04	0.04
SFA	17.62	17.02	4.00	5.22
MUFA	10.19	11.84	6.17	8.02
PUFA	14.98	21.11	15.34	20.21

SM = Soybean Meal; SC = Soybean Cake; BW= Body Weight; SFAs = Saturated Fatty Acids; MUFAs = Monounsaturated Fatty Acids; PUFAs = Polyunsaturated Fatty Acids

Table S2: growth performance of growing-finishing pigs.

**Group**		**SM**	**SC**	**EMS**	***p-Value***
Pens	nr	8	8	-	
BW d1	Kg	50.1	50.9	29.09	0.78
BW d113	Kg	120.2	124.2	100.5	0.44
BW d186	Kg	164.7	169.6	92.0	0.36
ADG d1-113	g/d	621	649	4315	0.39
ADG d113 -186	g/d	610	621	8322	0.81
ADG d1-186	g/d	616	638	2486	0.40
FCR 1-113d	kg/kg	2.54	2.33	0.094	0.19
FCR 113 -186d	kg/kg	4.37	4.36	0.490	0.98
FCR 1-186d	kg/kg	3.70	3.51	0.110	0.29

SM = Soybean Meal; SC = Soybean Cake; BW= Body Weight; ADG=Average Daily Gain; FCR=Feed Conversion Ratio; EMS = Error Mean Square
